# Massive irreparable rotator cuff tear with deltoid tear managed with reverse total shoulder arthroplasty: Case report and review of literature

**DOI:** 10.1097/MD.0000000000044507

**Published:** 2025-10-03

**Authors:** Jianan Liu, Cheng Luo, Yanjuan Chen, Yijun He, Jiongfeng Huang

**Affiliations:** a Department of Osteoarthropathy and Sports Medicine, Affiliated Panyu Central Hospital, Guangzhou Medical University, Guangdong, P. R. China.

**Keywords:** case report, deltoid tear, irreparable rotator cuff tear, reverse total shoulder arthroplasty, rotator cuff tear arthropathy

## Abstract

**Rationale:**

Massive irreparable rotator cuff tear (IRCT) with concurrent deltoid tear poses a therapeutic challenge, as the deltoid compensates for lost rotator cuff function. Reverse total shoulder arthroplasty is often contraindicated, but emerging evidence supports its use with deltoid repair, even in patients with prior radiotherapy. This case addresses the knowledge gap in managing such complex cases.

**Patient concerns:**

A 67-year-old right-hand dominant male reported chronic right shoulder pain worsening after a fall, with limited range of motion (active flexion 40°, abduction 40°, extension 30°), a palpable middle deltoid gap, and supraspinatus/infraspinatus wasting. History included oral carcinoma resection, chemotherapy, and radiotherapy 3 years prior, without metastasis.

**Diagnoses:**

Massive IRCT of supraspinatus, infraspinatus, and subscapularis with retraction (Goutallier grade 3, Hamada 4b); 4-cm retracted middle deltoid tear; rotator cuff tear arthropathy with superior humeral head migration, acromial sclerosis, and glenohumeral degeneration, confirmed by x-ray, computed tomography, and magnetic resonance imaging. Preoperative Constant-Murley score: 27 (pain: 2, activities of daily living: 10, movement: 12, strength: 2).

**Interventions:**

One-stage reverse total shoulder arthroplasty with deltoid repair via deltopectoral incision extended along the anterolateral acromion. Deltoid stump reattached to acromion using transosseous sutures. Rehabilitation: 6 weeks immobilization at 90° abduction, followed by passive, active-assisted, and active range of motion exercises.

**Outcomes:**

At 1-year follow-up, pain resolved; active abduction/flexion improved to 165°, extension to 30°, with pain-free rotations. Postoperative Constant-Murley score: 93 (pain: 15, activities of daily living: 20, movement: 40, strength: 18). X-rays showed stable prosthesis without loosening or instability.

**Lessons:**

Reverse total shoulder arthroplasty with deltoid repair can achieve favorable short-term outcomes in IRCT with deltoid compromise, challenging traditional contraindications. Key factors include preserved anterior/posterior deltoid function, precise surgical techniques, and rehabilitation compliance. Long-term studies are needed for validation.

## 1. Introduction

Reverse total shoulder arthroplasty (RTSA) has become prevalent for patients with irreparable rotator cuff tear (IRCT) or rotator cuff tear arthropathy (RCTA). Its inverted articulation not only creates a fixed fulcrum that prevents superior migration of the humeral head but also alters shoulder biomechanics by medializing and distalizing center of rotation, thus lengthening the moment arm of the deltoid, making it the primary driver of shoulder motion.^[1]^ Therefore, it is generally recognized that a functional deltoid is integral to a successful RTSA. However, in RCTA, the humeral head shifts superior without a rotator cuff, causing the greater tuberosity to rub against the deltoid muscle. It is speculated that the constant friction causes the deltoid to thin and eventually rupture.^[2]^ The coexistence of RCTA and deltoid muscle tear or impairment presents a challenging clinical dilemma, as concerns have been raised about instability, limited function, and potential failure after RTSA where the deltoid is compromised.

When the rotator cuff tear is deemed irreparable, there are several alternatives, such as arthrodesis, resection arthroplasty, tendon transfer and even conservative treatments.^[3]^ They are reserved when RTSA is not a viable option, when taking into account the factors such as patient age, comorbidities and outcome expectations. While these options might possibly alleviate pain and improve functionality to some extent, they often fall short of restoring the shoulder to the pre-tear function and are associated with their own set of limitations.

Despite the growing body of literature on RTSA for IRCT and RCTA,^[4]^ there is still a lack of evidence addressing the choice and outcomes in patients with concomitant deltoid tears. This knowledge gap necessitates the further exploration of surgical decision-making. In this report, we present a case of massive IRCT with concomitant deltoid tear managed with RTSA that showed improvement in pain and range of motion (ROM), highlighting the rationale of clinical decision-making, surgical techniques and outcomes. Our case adds to the emerging literature that challenges the notion of ruling out RTSA in the setting of deltoid compromise, highlighting the importance of customized surgical decision-making and postoperative rehabilitation strategies. We also review the existing literature to provide context and guidance for managing similar cases.

## 2. Case presentation

**Figure 1.**
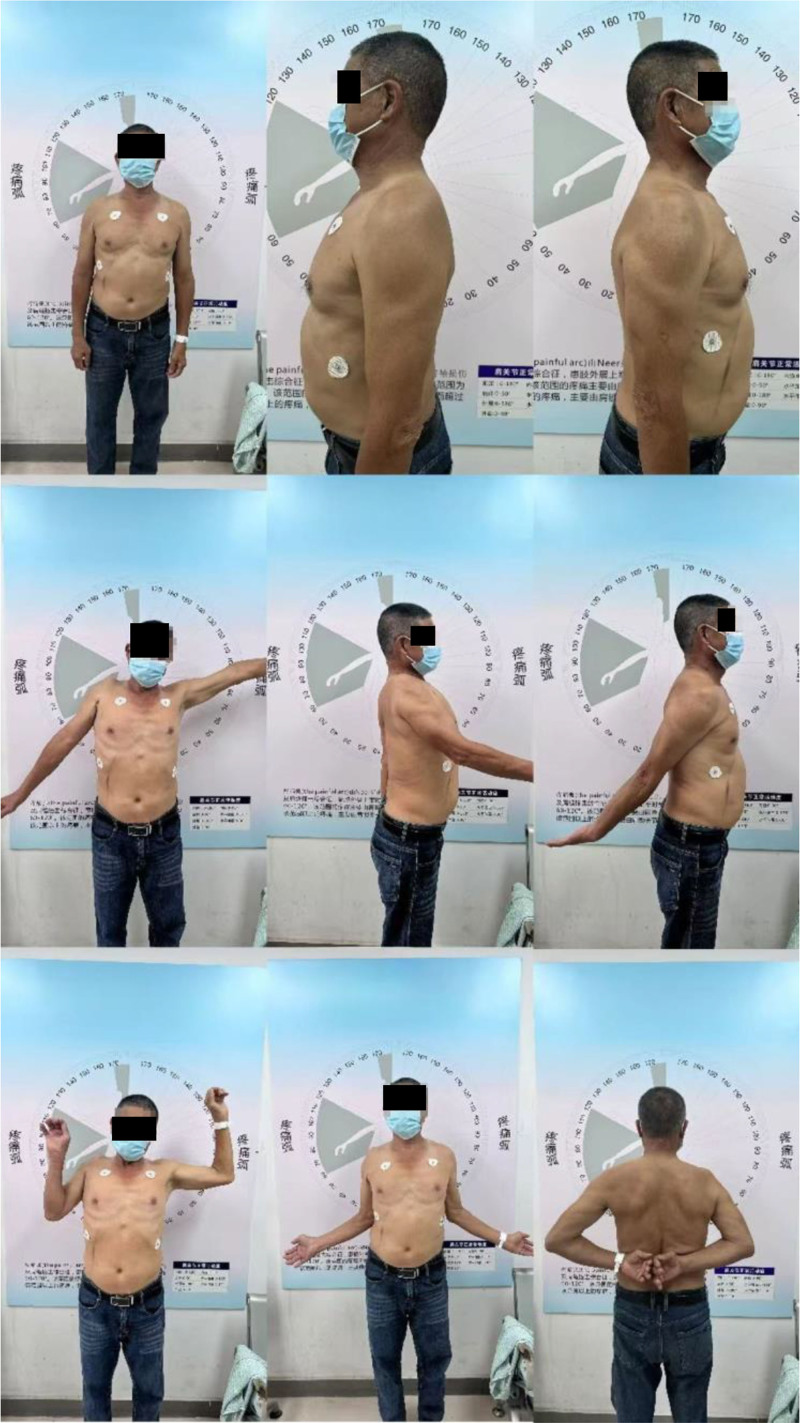
Preoperative shoulder range of motion. Photographs demonstrating significant limitations in active forward flexion (approximately 40°), abduction (approximately 40°), and external/internal rotation before surgical intervention.

**Figure 2.**
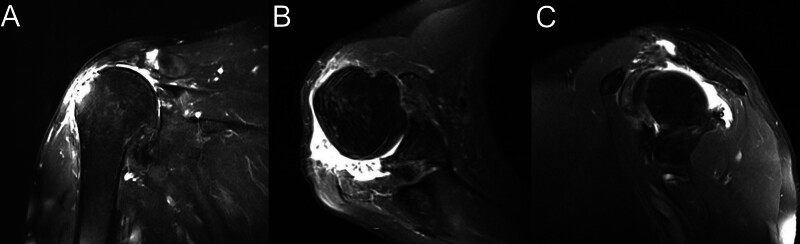
Preoperative magnetic resonance imaging. (A) Coronal T2-weighted image showing superior migration of the humeral head, significant fatty infiltration (Goutallier grade 3) and retraction of the supraspinatus tendons, and degenerative changes consistent with rotator cuff tear arthropathy (Hamada 4b). A retracted tear of the middle deltoid origin from the acromion (arrow) is also evident. (B) Transversal T2-weighted image further illustrating the retraction of infraspinatus tendon and the massive rotator cuff tear. (C) Axial T2-weighted image further demonstrating the irreparable rotator cuff tear.

A 67-year-old right-hand dominant male presented with chronic mild right shoulder pain and limited ROM with progressive worsening 1 month after a fall. Physical examination revealed a palpable gap in the middle deltoid region, significant wasting of supraspinatus and infraspinatus muscles, with limited active forward flexion of 40°, extension of 30° and abduction of 40° (Fig. 1), with unremarkable neurovascular evaluation. The patient had a history of oral carcinoma resection and lymph node dissection 3 years prior and received the following chemotherapy and radiotherapy. Radiology and blood workups showed no evidence of metastasis. X-ray and computed tomography scan revealed superior migration of the right humeral head, with sclerotic changes in the acromion. Magnetic resonance imaging scan (Fig. 2) revealed significant tears and retraction of the supraspinatus, infraspinatus and subscapularis tendon, and a tear of the middle deltoid with retraction of 4cm from the acromion (Goutallier grade 3, Hamada 4b). Degenerative changes in glenohumeral cartilage were also noted. The patient’s Constant-Murley score was 27 points, comprised of 2 points for pain, 10 points for activities of daily living, 12 points for movement, and 2 points for strength.

**Figure 3.**
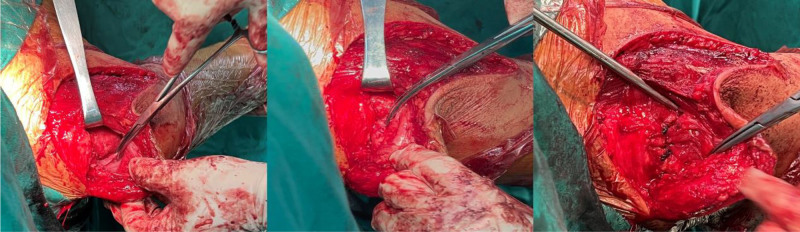
Intraoperative demonstration before and after deltoid repair. (Left and middle) Exposure of the retracted tendinous stump of the middle deltoid (held by forceps) after mobilization, before reattachment. (Right) Appearance after the deltoid stump has been reattached to the lateral acromion using transosseous sutures (sutures visible).

**Figure 4.**
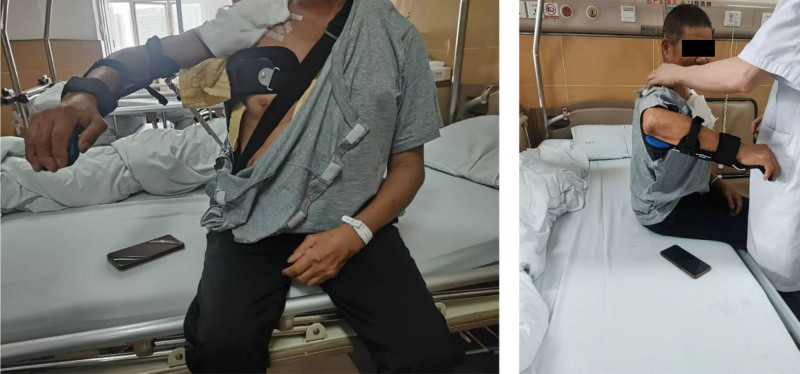
Splint immobilization demonstration. Photograph showing the patient wearing the shoulder abduction splint, which maintained the operative arm in approximately 90° of abduction for the initial 6 weeks postsurgery to protect the deltoid repair.

The 1-staged RTSA (Comprehensive^®^ Reverse Shoulder System, Zimmer Biomet, Inc., Warsaw) with deltoid repair was selected for this case. The patient was positioned in a semi-beach chair position following general anesthesia and regional block. A deltopectoral approach was used, with proximal incision extending about 4-cm along the lateral broader of acromion. Intraoperative findings revealed the tear and retraction of supraspinatus, infraspinatus and subscapularis tendon. Additional wear of humeral cartilage was also noted. Intramedullary resection of the humeral head was performed followed by glenoid preparation. A 25-mm baseplate with a 36-mm glenosphere was positioned low on the glenoid and secured by 4 peripheral screws. An 11-mm humeral stem was impacted at 30° retroversion followed by the placement of the bearing. After confirming stability in all planes of motion, the remainder was the deltoid repair. The tendinous portion of the torn deltoid was meticulously preserved, and a soft tissue release was performed from proximal to distal around the middle deltoid. With the affected shoulder abducted at 90°, the torn stump could be approximated to the acromion without tension. Next, 2 transosseous tunnels were created in the acromion. The sclerotic bone on lateral edge was partially removed, and the stump was reattached side-to-side using simple interrupted suture with #1 Ethibond (Fig. 3). Marginal conversion of the fascia over anterior and middle deltoid was also performed. Postoperatively, the patient showed no neurovascular deficits. The affected shoulder was strictly immobilized at 90° abduction in a splint for 6 weeks while active mobilization of the elbow, wrist, and fingers was permitted (Fig. 4). Following splint removal, gradual passive ROM exercises were initiated for 2 weeks. The patient progressed to active-assisted shoulder motion exercises for an additional 2 weeks and then full active motion exercises.

**Figure 5.**
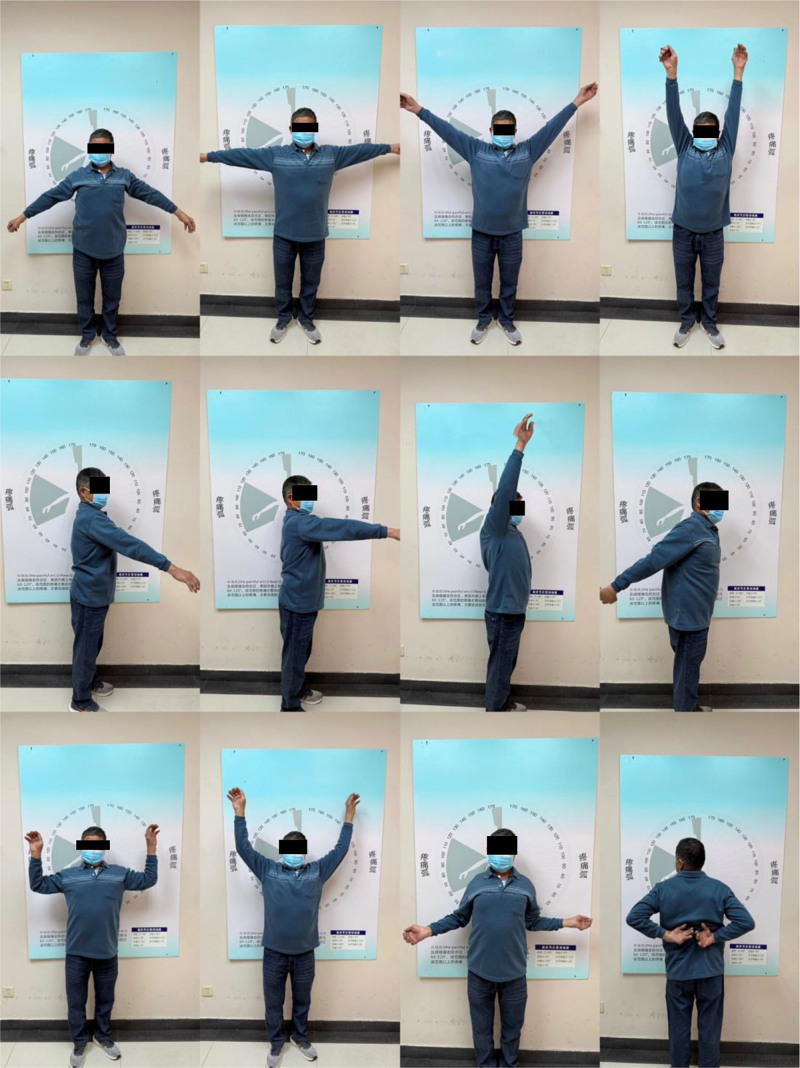
Postoperative shoulder range of motion. Photographs showing substantial improvement, with pain-free active forward flexion (approximately 165°), abduction (approximately 165°), and functional external/internal rotation.

**Figure 6.**
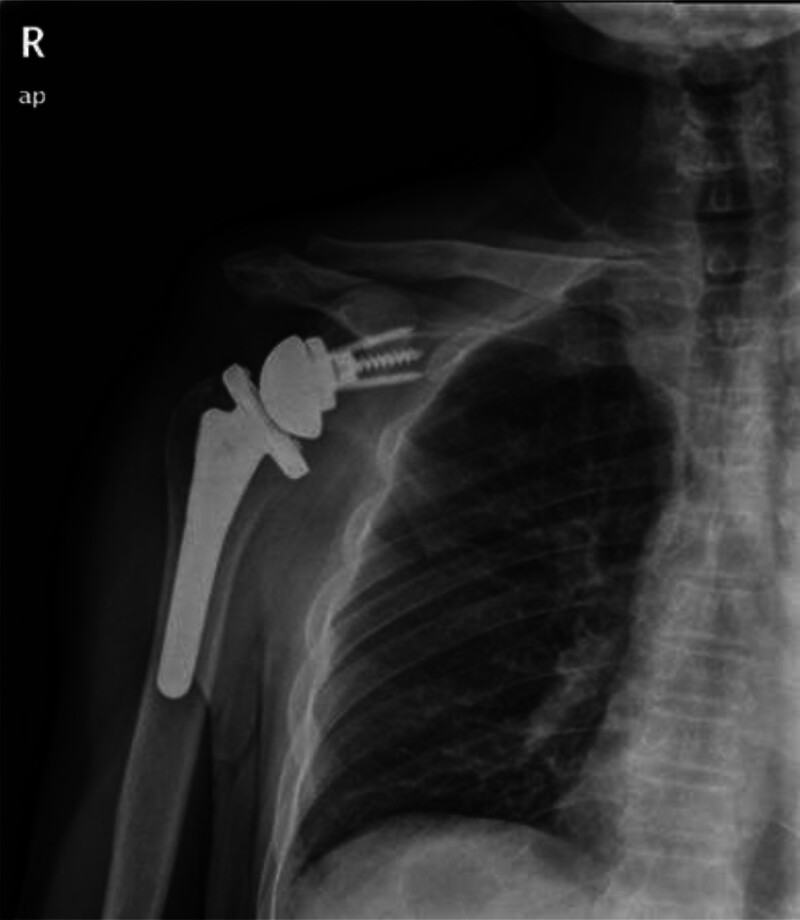
Postoperative X-ray follow-up at 1 year. The image shows the reverse total shoulder arthroplasty components (glenosphere, baseplate, humeral stem, and bearing) are well-positioned and appropriately fixed, with no radiographic signs of loosening, subsidence, or significant scapular notching.

At the 1-year follow-up visits, the patient reported pain-free ROM, with active abduction of 165°, flexion of 165°, extension of 30° and pain free external and internal rotation (Fig. 5). Postoperative x-ray showed correct placement of the prosthesis without scapular notching, fractures and signs of component loosening (Fig. 6). The patient had a postoperative Constant-Murley score of 93, comprising 15 points for pain, 20 points for activities of daily living, 40 points for movement, and 18 points for strength.

## 3. Discussion

Deltoid tears, when associated with IRCT, can cause significant shoulder dysfunction and pain. The deltoid muscle compensates for shoulder abduction in the presence of IRCT. It is reported that spontaneous deltoid tears, often involving the middle third, can occur in the setting of chronic rotator cuff tears.^[5,6]^ Deltoid tears can also result from iatrogenic causes including previous shoulder surgery or repeated steroid injections.^[6,7]^ In the context of our case, the patient had a history of radiotherapy, a traumatic event and a chronic rotator cuff tear, all of which might have contributed to the deltoid tear. Research indicates that the middle deltoid’s contribution to abduction strength increases proportionally with the angle of abduction, accounting for 24% at the neutral position and up to 75% at 120° of abduction.^[8]^ In our patient, the anterior and posterior deltoid muscles remain intact, which likely explains the preservation of approximately 40° of shoulder abduction through compensatory mechanisms. Additionally, neurovascular examination results were unremarkable. Given the patient’s acute onset of abduction weakness following a fall, consistent with a middle deltoid tear: we opted for a surgical repair strategy. Notably, we selected RTSA despite the conventional emphasis on the need for a fully functional deltoid. We performed a 1-stage RTSA and repaired the deltoid by reattaching the tendinous stump to the acromion with transosseous tunnels by side-to-side suturing. Postoperatively, the patient demonstrated significant improvements in pain relief and ROM at both 3-month and 1-year follow-up, with no evidence of shoulder instability or component loosening.

To date, limited literature have attempted to perform RTSA with or without deltoid repair. But some studies yield favorable outcomes that challenge the conventional understanding (Table 1).^[6,9–15]^

**Table 1:** Summary of existing literature on IRCT concomitant with deltoid deficiency.

Team of study	Year	Number of cases	Patient cohort	Treatment options	Outcomes	Key findings
Morisawa et al^[9]^	1997	2	Patients with spontaneous rupture of the deltoid muscle associated with massive rotator cuff tear	One case receiving an open deltoid repair and tendon transfer using subscapularis and infraspinatus tendons to replace the torn supraspinatus and partial infraspinatus	Not reported	Highlighting the possibility of spontaneous deltoid rupture in the setting of massive rotator cuff tears
Glanzmann et al^[10]^	2009	1	Patient with a pseudoparalytic shoulder with rotator cuff tear arthropathy after failed latissimus dorsi transfer for the prior rotator cuff tear	RTSA without deltoid repair	Improved shoulder function, with an absolute Constant score improving from 22 to 62 points 2 yr after reconstruction	RTSA only can be a successful salvage procedure for patients with RCTA and deltoid deficiency
Tay and Collin^[11]^	2011	1	Patient with irreparable spontaneous deltoid rupture in rotator cuff arthropathy	RTSA without deltoid repair	Good outcome. At 1 yr, the patient achieved active forward flexion of 140°, abduction of 120°, and external rotation of 40°. The Constant score improved from 18 preoperatively to 75	RTSA can be successful in patients with irreparable deltoid rupture and RCTA without the need for direct deltoid repair
Lädermann et al^[12]^	2013	49	Patients with various etiologies of deltoid impairment, including post-traumatic, postoperative, axillary nerve lesion, resection or muscular flap transfer, postradiation, and deltoid avulsion	RTSA without deltoid repair	Improved forward elevation and Constant-Murley scores	RTSA can lead to satisfactory functional outcomes in patients with preoperative deltoid impairment; less satisfactory in cases of global deltoid impairment
Schneeberger et al^[13]^	2014	19	Patients with failed deltoid flap transposition for IRCT	RTSA without deltoid repair	RTSA could improve function with patient variations; all patients regained anterior active elevation above 90°; some patients had persistent weakness	37% of patients experiencing complications, requiring 16 revision surgeries. Complications included peri-prosthetic fracture, infections, polyethylene component disassembly, bony spur issues, instability and aseptic loosening of the glenoid component
Garofalo et al^[6]^	2016	18	Patients with IRCT and associated dehiscence or rupture of the anterior and middle deltoid muscle. Four patients had previous shoulder surgery, and 14 patients had attritional tears	RTSA with deltoid repair	Mean active anterior elevation improved from 53 ± 9.1 to 132.7 ± 11.6; active external rotation improved from 22.4 ± 3.6 to 47.8 ± 5.8	Supporting the feasibility and potential benefits of reverse shoulder arthroplasty combined with deltoid repair in patients with massive irreparable rotator cuff tears and associated deltoid tears
Dosari et al^[14]^	2017	1	Patient with a gunshot extensive injury to the shoulder with a massive rotator cuff tear, severe osteoarthritis, and deltoid deficiency	RTSA without deltoid repair (not applicable)	Tolerable pain on exertion and no pain at rest 1 yr after the operation. Passive pain-free full range of motion but no progress in active range of motion beyond that upon discharge	Without functional deltoid, RTSA can provide pain relief in patients with significant shoulder injuries, but active functional gains may be limited
Oh et al^[15]^	2022	2	Patients with massive rotator cuff tear accompanied by a deltoid muscle rupture	RTSA with deltoid repair	Good clinical outcomes	Supporting the feasibility and potential benefits of reverse shoulder arthroplasty combined with deltoid repair in patients with massive irreparable rotator cuff tears and associated deltoid tears

IRCT = irreparable rotator cuff tear, RCTA = rotator cuff tear arthropathy, ROM = range of motion, RTSA = reverse total shoulder arthroplasty.

Several studies and case reports have explored the outcomes of RTSA without deltoid repair, highlighting RTSA might be a viable or salvaging resort in managing deltoid-deficient RCTA. Glanzmann et al^[10]^ published a case report detailing the use of RTSA after a failed deltoid muscle flap transposition. The postoperative outcome was described satisfactory with an improvement in the patient’s Constant score from 22 to 62 points 2 years after the surgery. In a similar cohort of 19 patients, Schneeberger et al^[13]^ found that RTSA without deltoid repair could improve function but varied among patients, with some having persistent weaknesses. The study also suggested that a circumscribed anterolateral defect of the deltoid muscle might result in diminished strength after RTSA. The complication rate was high (37%) in the series including peri-prosthetic fracture, infections, polyethylene component disassembly, bony spur issues, instability and aseptic loosening of the glenoid component. Tay and Collin^[11]^ provided a single case that RTSA can be successful even with an irreparable deltoid rupture, demonstrating a good outcome at 1 year’ follow-up. A larger cohort with 49 patients was studied by Lädermann et al,^[12]^ with varying degrees and etiologies of deltoid impairment prior to surgery. These etiologies included post-traumatic, postoperative, axillary nerve lesion, resection or muscular flap transfer, postradiation, and deltoid avulsion. Through either the deltopectoral or the trans-deltoid approach, RTSA was performed. Postoperatively, improved forward elevation and Constant-Murley scores were noted. However, cases involving gunshot, complete avulsion or axillary nerve palsy showing global deltoid impairment were less satisfactory. In a separate case, Dosari et al^[14]^ reported a failed latissimus dorsi flap transfer due to previous gunshot injury resulting in a massive rotator cuff tear, severe osteoarthritis, and deltoid deficiency. RTSA was performed, although a deltoid repair was not feasible. The patient reported tolerable pain on exertion, no pain at rest, but limited active function after 1 year.

Garofalo et al^[6]^ studied a cohort of 18 patients with IRCT and an associated tear or dehiscence of the anterior and middle deltoid muscle. Among these patients, 4 had iatrogenic tears from previous surgery, while 14 had attritional deltoid tears. All patients underwent RTSA via a modified anterosuperior approach. The anterior and middle fibers of the deltoid were mobilized and sutured back to their origin on the acromion using a side-to-side and transosseous sutures. The outcomes demonstrated significant improvements in ROM. Likewise, Oh et al^[15]^ demonstrated similar outcomes in 2 cases with RTSA and deltoid repair. These studies acknowledge RTSA with concomitant deltoid repair is a viable treatment option considering the fact that an intact or functional anterior and middle deltoid is crucial for a well-balanced arthroplasty. Collectively, the literature suggests RTSA may provide pain relief even with deltoid compromise, but functional outcomes are variable and depend heavily on residual deltoid function and the possibility of repair

Overall, these findings suggested that RTSA, when combined with or without simultaneous deltoid repair, could yield favorable outcomes in select patients previously deemed to have an absolute contraindication for shoulder arthroplasty. In the context of deltoid tear and repair, several factors must be considered: the extent, location, and repairability of the deltoid tear; the presence of residual deltoid function; and the etiology of the tear, such as degenerative, traumatic, or neurogenic causes.

Although neck radiotherapy is known to impair soft tissue healing and reduce bone quality, published reports indicate that outcomes following shoulder arthroplasty after external-beam radiation have been largely positive. These studies demonstrate satisfactory tendon integrity and implant survival, with only a moderate increase in complication rates compared to nonirradiated controls.^[16–19]^ Additionally, a case report described a pathological scapular fracture induced by radiation therapy following RTSA, underscoring the potential for compromised bone integrity and impaired healing in irradiated tissues.^[20]^ Notably, there have been no documented cases of deltoid repair failure. In the present patient, the deltoid muscle was located outside the high-dose radiation field; it was successfully repaired and maintained under minimal tension during the early postoperative period. At the 1-year follow-up, the repair remains intact, without signs of implant loosening. Consequently, we believe that follow-ups are warranted to identify deltoid repair integrity and long-term prosthesis stability related to radiation exposure.

In our case, another distinctive feature is our use of the conventional deltopectoral approach, which includes extending the proximal incision along the lateral edge of the acromion to mobilize and repair the torn deltoid. Compared to the trans-deltoid approach, our method offers direct exposure either for the arthroplasty and the deltoid repair, while minimizing the risk of iatrogenic injury to the axillary nerve that is crucial to deltoid’s function.

In addition to technical considerations, patient-specific factors should also be considered for the successful outcome. The patient demonstrated strict compliance during the 90°-immobilization period and consistently performed home-based rehabilitation exercises afterward. Maintaining the shoulder in 90°-abduction postoperatively was intended to minimize tension on the repaired deltoid, facilitating optimal healing conditions. This position reduces stress on the repair site and supports proper alignment during the critical early healing phase. Based on the patient’s history, it was likely that a rotator cuff deficiency existed prior to the traumatic fall, but shoulder function was largely compensated by the deltoid. Furthermore, the patient’s own physique is likely to contribute to faster recovery.

While this case report highlights the successful management of an IRCT with concomitant deltoid tear using RTSA and deltoid repair, it is not without limitations. The single-case design inherently limits the generalizability of the findings, as outcomes may vary in patients with different etiologies, such as global deltoid paralysis or neurogenic tears. Notably, the relatively short follow-up period of 1 year precludes assessment of long-term complications, such as prosthetic loosening, deltoid re-tear. Lastly, while the patient demonstrated exceptional compliance and a strong pre-injury physical condition, these factors may not be replicable in all cases, potentially limiting the broader applicability of the reported outcomes. Therefore, larger, long-term studies to validate the findings and refine surgical decision-making in this complex patient population is needed.

## 4. Conclusion

This case demonstrates that 1-stage RTSA with deltoid repair can be a viable management strategy for patients with massive IRCT and concomitant deltoid compromise. Despite the traditionally held notion that a fully functional deltoid is integral for successful RTSA, our patient achieved considerable pain relief and functional gains at short-term follow-up. The availability of sufficient anterior and posterior deltoid function, meticulous surgical repair of the middle deltoid, and conscientious postoperative rehabilitation were key factors in this favorable outcome. Further long-term studies and larger cohorts are warranted to validate and refine the indications, surgical techniques, and postoperative protocols in this nuanced patient population.

## 5. Patient perspective

I had reached a point where shoulder pain was affecting not only my physical activities but also my overall mood and quality of life. This surgery, although complex, gave me hope for improvement. After the surgery, the immobilization period was difficult as I had to rely on others for simple tasks, but the gradual shift to passive and then active exercises brought noticeable improvements. My pain diminished, and as my shoulder regained mobility. I appreciated the team offering me the treatment that helped me regain activities I once thought were impossible.

## Author contributions

**Conceptualization:** Cheng Luo, Yijun He, Jiongfeng Huang.

**Data curation:** Yanjuan Chen, Yijun He.

**Funding acquisition:** Cheng Luo, Yijun He.

**Investigation:** Cheng Luo, Yanjuan Chen.

**Methodology:** Yanjuan Chen.

**Project administration:** Yijun He, Jiongfeng Huang.

**Supervision:** Yijun He, Jiongfeng Huang.

**Writing – original draft:** Jianan Liu.

**Writing – review & editing:** Yijun He.
